# Genomic Characterization and Virulence Potential of Two *Fusarium oxysporum* Isolates Cultured from the International Space Station

**DOI:** 10.1128/mSystems.00345-18

**Published:** 2019-03-19

**Authors:** Camilla Urbaniak, Peter van Dam, Alexander Zaborin, Olga Zaborina, Jack A. Gilbert, Tamas Torok, Clay C. C. Wang, Kasthuri Venkateswaran

**Affiliations:** a Jet Propulsion Laboratory, California Institute of Technology, Pasadena, California, USA; b Molecular Plant Pathology, University of Amsterdam, Amsterdam, the Netherlands; c University of Chicago, Chicago, Illinois, USA; d Lawrence Berkeley National Laboratory, Berkeley, California, USA; e University of Southern California, Los Angeles, California, USA; University of Trento

**Keywords:** *Fusarium*, International Space Station, fungi, genomics

## Abstract

Two isolates of Fusarium oxysporum, ISS-F3 and ISS-F4, were cultured from the dining table on the International Space Station (ISS). Genomic analyses using EF-1α sequences, presence/absence of effector proteins, k-mer comparisons, and single nucleotide polymorphisms indicate that these two strains are genomically different from 65 known sequenced strains. Functional analysis revealed that ISS-F3/F4 had higher relative abundances of polyketide synthase domains than a non-plant-pathogenic soil isolate, used for biocontrol properties (Fo47), and a clinical isolate (FOSC-3a). Putative secondary metabolite analysis indicates that ISS-F3/F4 may produce yet-unreported polyketides and nonribosomal peptides. While genomic analysis showed that these ISS strains are unlikely to be plant pathogens, a virulence assay using an immunocompromised Caenorhabditis elegans model of fusariosis revealed that they were virulent and may represent opportunistic pathogens in animals, including humans. However, its effects on the health of immunocompromised humans warrant further study.

**IMPORTANCE** This is the first study to isolate and characterize F. oxysporum isolates from a built environment, as well as one that has been exposed to space. The characterization and analysis of these two genomes may have important implications for the medical, agricultural, and food industries as well as for the health of the crew who coinhabit the ISS with these strains.

## Abstract (precis)

This is the first study to isolate and characterize F. oxysporum isolates from a built environment, as well as one that has been exposed to space. The characterization and analysis of these two genomes may have important implications for the medical, agricultural, and food industries as well as for the health of the crew who coinhabit the ISS with these strains.

## INTRODUCTION

Fusarium oxysporum is a presumed asexual, filamentous fungus ubiquitous in soil and infamous for causing vascular wilt and root rot disease in many economically important plants and crops (1). While the number of hosts this species can infect is extensive (tomatoes, bananas, cotton, strawberries, eggplants, tulips, soybean, beets, and chrysanthemum, to name only a few) (2), individual isolates show a high degree of host specificity and are assigned to *formae speciales* (f. sp.) based on the host it can infect (3). For example, F. oxysporum f. sp. *cubense* causes fungal wilt in bananas (“Panama disease”) while f. sp. *melonis*, f. sp. *tulipae,* and f. sp. *cucumerimum* infect and cause disease in melons, tulips, and cucumbers, respectively. At present, there are approximately 150 *formae speciales* (i.e., host-specific groups of strains) that have been described (2).

While well known as a phytopathogen, F. oxysporum can also cause infections in humans, ranging from superficial and localized (skin, nail, cornea, and superficial wound) in immunocompetent individuals to invasive and disseminated (blood culture, deep tissue involvement, or isolation of strain from two or more body sites) in immunocompromised patients (4–6). Since members of the fungal genus *Fusarium* are intrinsically resistant to most available antifungals on the market (7), prognosis is often poor and sometimes fatal for those afflicted with systemic fusariosis (6, 8).

Fungi are known to produce many bioactive molecules that can benefit humans, such as antimicrobials, antialgals, immunosuppressants, compounds cytotoxic to cancer cells, insecticides, and antioxidants (9–11), and F. oxysporum is no exception. F. oxysporum isolated from the plants Smallanthus sonchifolius and Catharanthus roseus were shown to produce, in appreciable amounts, the anticancer drugs anhydrofusarubin and beauvericin (12) and vinblastine and vincristine (13), respectively. An isolate of F. oxysporum cultured from Juniperus recurva was shown to produce the aryltetralin lignin podophyllotoxin (14), which is a precursor for the chemical synthesis of anticancer drugs like etoposide, teniposide, and etopophose (15, 16). Podophyllotoxin is also known for its antiviral (17) and antiparasitic (18) activity.

In an ongoing microbial tracking study of the International Space Station (ISS), two F. oxysporum isolates were cultured from the dining table in the U.S. module of the ISS. The genomes of these two isolates were sequenced and compared to 65 other F. oxysporum isolates. The predicted proteins were annotated and mined for the presence of secondary metabolites and putative virulence factors. The virulence of these isolates in an immunocompromised (MAPKK-deficient) Caenorhabditis elegans model of invasive fusariosis was also assessed. This is the first study to isolate and characterize F. oxysporum isolates from a built environment as well as one that has been exposed to space.

## RESULTS

Two *F. oxysporum* isolates (designated ISS-F3 and ISS-F4) were cultured from the dining table of the U.S. module of the ISS. The genomes were paired-end sequenced (2 × 100 bp) on the Illumina HiSeq platform with a 350-bp insert size, resulting in 48 million (ISS-F3) and 42 million (ISS-F4) reads. These paired-end reads were *de novo* assembled into scaffolds (k-mer size = 86) using ABySS (version 2.0.2) (19). The assembled genome size for ISS-F3 was 53.6 Mb and for ISS-F4 was 53.8 Mb, which is comparable to the genome size of the model strain, F. oxysporum f. sp. *lycopersici* strain 4287 (59.9 Mb) (20). Table 1 provides general assembly statistics of the draft genomes of F. oxysporum ISS-F3 and ISS-F4.

**TABLE 1:** General assembly statistics for the draft genomes of Fusarium oxysporum ISS-F3/F4 strains

Isolate	Read count (million)	k-mer size	*N*_50_ (bp)	No. of scaffolds (total)	No. of scaffolds over 1 kb	Max scaffold length (Mb)	Genome size (Mb)	GC content (%)	Coverage (×)
ISS-F3	48	86	923,684	6,166	736	3.24	53.6	47.05	90
ISS-F4	42	86	940,233	7,066	948	2.23	53.8	46.88	80

### Phylogenetic relationship of ISS-F3/F4 among different F. oxysporum f. sp.

**FIG 1:**
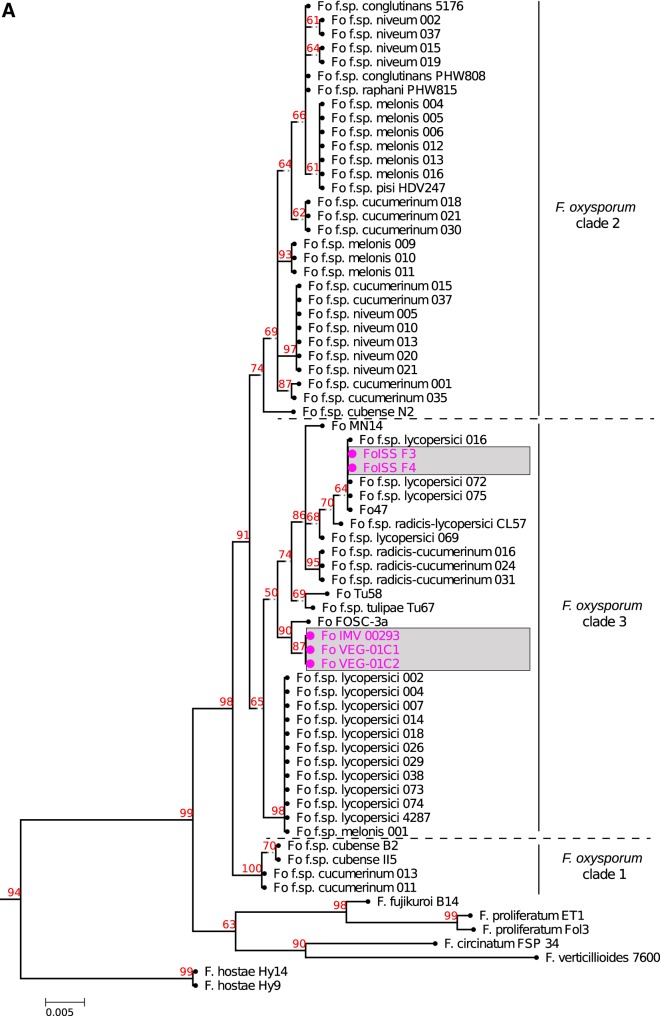

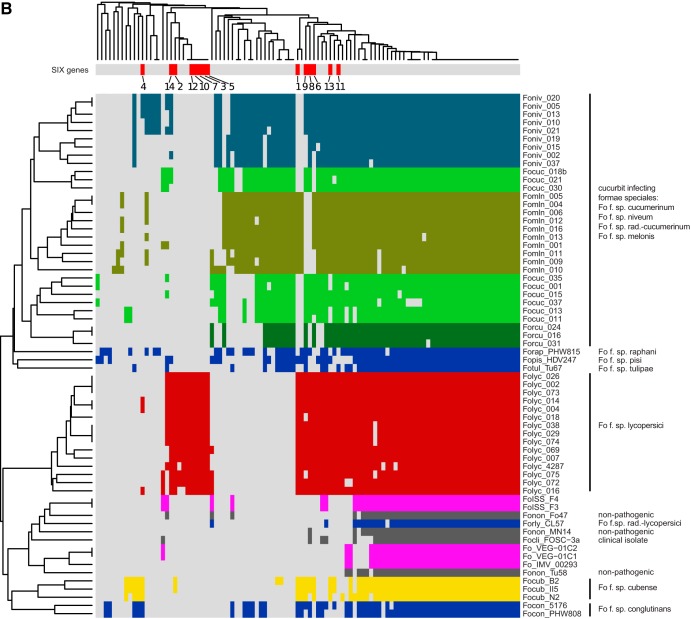
Phylogenetic relationship of ISS-F3/F4 among other F. oxysporum isolates. (A) The phylogenetic tree constructed from EF-1α sequences of 62 F. oxysporum isolates places ISS-F3/F4 in clade 3, belonging to a clonal lineage shared by lycopersici_016, lycopersici_072, lycopersici_075, and Fo47. Other *Fusarium* species (bottom) were included to root the tree. The bootstrap confidence (in percent; 1,000 replicates performed) is shown in red numbers in the tree. (B) Presence/absence profiles of ISS-F3/F4 with 62 other strains based on 104 candidate effector proteins. Colored boxes indicate the presence of a particular effector gene, with gray indicating its absence in the genome. The list of effector genes is shown in Data Set S4. *Formae speciales* that cause disease in the same host cluster together, as seen by the groupings of green (cucurbit-infecting strains), red (tomato-infecting strains), and yellow (banana-infecting strains). Dark gray represents non-plant-pathogenic isolates, and pink represents strains isolated from or flown to the ISS. Blue represents other plant pathogens. ISS-F3/F4 contained relatively few effector genes compared to the number found in the plant pathogens (green, red, yellow, and blue) and showed the most similar pattern (number and type) to Fo47, a non-plant-pathogenic soil isolate with a biocontrol function.

The conserved marker gene translation elongation factor 1 alpha (EF-1α) was used to determine the phylogenetic relatedness of ISS-F3/F4 to 62 other F. oxysporum strains, belonging to various *formae speciales,* and seven strains belonging to other *Fusarium* species included as outgroups, based on clonal (asexual) vertical inheritance. EF-1α allows for phylogenetic species recognition because it is applicable across *Fusarium* and it is informative at or near the species level and orthologous across the genus (21). Based on EF-1α phylotyping, F. oxysporum can be divided into 3 distinct clades with various clonal lineages within each clade (22, 23). The phylogenetic tree inferred in Fig. 1A clusters ISS-F3/F4 into clade 3, belonging to a clonal lineage closely related to 4 other strains: three members of F. oxysporum f. sp. *lycopersici* and the non-plant-pathogenic biocontrol strain Fo47 (members of a clonal lineage have the same sequence of conserved genes). Three other isolates of interest are highlighted in Fig. 1A because they too had been isolated from harsh environments, two cultured from ISS-grown Zinnia hybrida plants that caused foliar, stem, and root rot (VEG-01C1 and VEG-01C2), and IMV00293, cultured at the site of the Chernobyl nuclear disaster 12 years after the explosion. The ISS *Z. hybrida* strains fell into a different branch from ISS-F3/F4 and were most closely related to IMV00293.

F. oxysporum genomes consist of a “core” set of housekeeping genes and an “accessory” genome which is not required for vegetative growth but contains most of, if not all, the genes that allow the fungus to invade and cause disease in a host plant (20). Utilizing 104 effector candidates, van Dam et al. were able to show that strains infecting the same host generally possess a similar set of effectors and that these effectors are often identical within a *forma specialis* (24). This indicates that the accessory genome, specifically the effector genes residing in these regions, can be used to identify *formae speciales* within the F. oxysporum species complex (FOSC), including newly encountered strains (24). Figure 1B shows the hierarchical clustering of presence/absence patterns of 104 effector sequences in ISS-F3/F4 along with 62 other strains. The effector profile of ISS-F3/F4 was the most similar to Fo47 (a non-plant-pathogenic soil isolate that has been shown to exert a biocontrol function) (25) with similarities to another non-plant-pathogenic isolate, MN25, and CL57 (NRRL 26381), an isolate causing root rot in tomato, also known as NRRL 26381. The ISS-F3/F4 strains do not contain as many effector genes as (almost) all of the plant pathogens examined and do not possess any of the SIX genes (see top panel of Fig. 1B), which encode secreted fungal proteins that were first discovered in the xylem sap of infected tomato plants and have a well-studied role in plant pathogenesis (26, 27).

### Relationship between ISS-F3 and ISS-F4.

Further analyses were conducted to determine how closely related ISS-F3 and ISS-F4 were to each other. The sequences of 10 phylogenetically informative loci (28) were compared between the two strains, and for all 10 loci, the sequences were identical between ISS-F3/F4. Second, a BLAST comparison was performed using contigs above 2 kb for each strain. Using ISS-F3 as the query, each of the 682 contigs matched a contig in ISS-F4 with either 99% or 100% identity with an E value of 0. Last, when single nucleotide polymorphism (SNP) analysis was performed against the reference genome Fo47, ISS-F3/F4 had the same single nucleotide variants. For this reason, it is believed that ISS-F3/F4 are clonal derivatives of each other.

### Strain identification.

Based on EF-1α phylogeny (Fig. 1A) and the presence/absence of effector proteins (Fig. 1B), ISS-F3/F4 appeared to be the most closely related to Fo47. To determine whether the ISS strains were Fo47 or represented a distinct strain, two *in silico* genomic comparisons were conducted.

### (i) k-mer comparison.

The KMC2 k-mer counter (29) was used to generate 50-bp long k-mers from 65 F. oxysporum genomes that were downloaded from GenBank (see Table S1 in Data Set S5 in the supplemental material). The k-mers generated from each genome were compared against the k-mers from all other genomes to find the ones that were specific to each of the 65 genomes analyzed. From all genome-specific k-mers that were identified, a subset was chosen from each genome to create the F. oxysporum k-mer database. These sequences were chosen based on number of occurrences (chosen if they appeared once, instead of multiple times within the genome) and the type of sequence (the sequence did not contain a lot of repeats and had a good balance of GC and AT ratios). The sequences making up this database are listed in Table S2.

10.1128/mSystems.00345-18.5 TABLE S2 F. oxysporum k-mer database. The sequences shown represent the 50-bp k-mer sequences that are unique to each strain and not present in the other 64 strains examined. Download Table S2, TXT file, 0.05 MB. Copyright © 2019 Urbaniak et al. 2019 Urbaniak et al. This content is distributed under the terms of the Creative Commons Attribution 4.0 International license.

The assembled genomes of ISS-F3/F4 were then compared using BLAST against the F. oxysporum k-mer database, and for ISS-F3/F4 to be considered one of the 65 F. oxysporum strains, every k-mer in the database belonging to a specific strain had to have a perfect match in the genome. For example, all k-mers generated for strain Fo47 in the database needed to match sequences in either the ISS-F3 or -F4 genome 100% (i.e., 50-bp k-mer/50-bp genome [no mismatches]) for ISS-F3 or -F4 to be considered Fo47.

None of the Fo47 unique k-mer sequences matched sequences in the ISS-F3 or ISS-F4 genome, and only 1 k-mer from strains *radicis-lycopersici* 26381 (CL57), UASWS AC1, *lycopersici* Fol074, and *melonis* Fom009 found an exact match. Based on the criteria above, ISS-F3/F4 were not the same strain as Fo47 nor any of the other 64 strains tested.

### (ii) Single nucleotide polymorphism (SNP) analysis.

**FIG 2:**
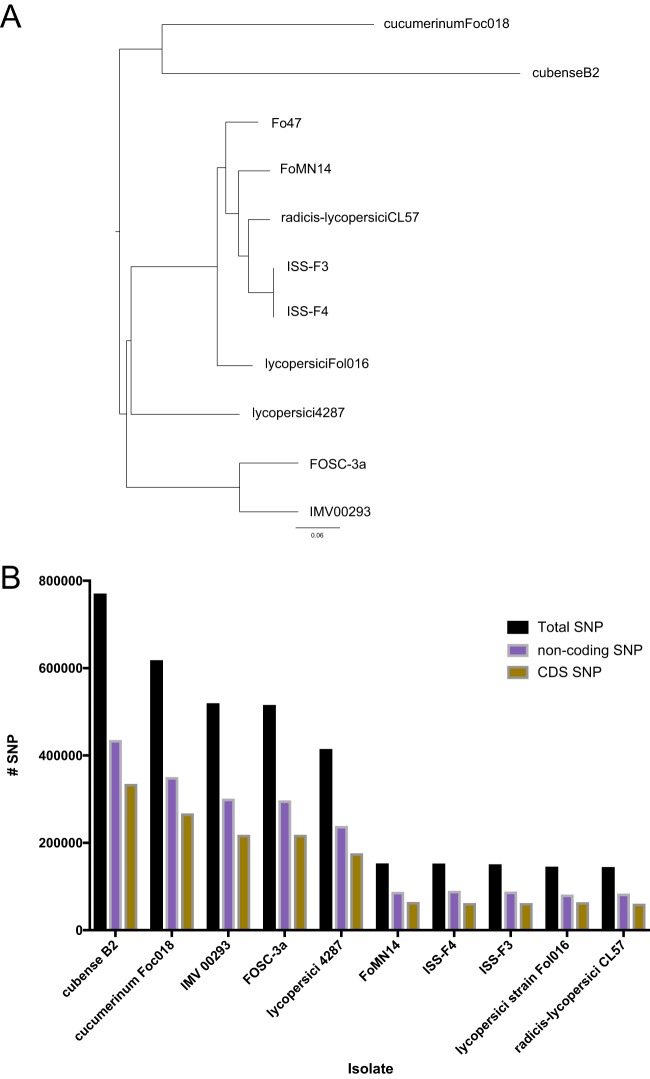
Single nucleotide polymorphism (SNP) analysis comparing ISS-F3/F4 to the reference genome Fo47. (A) Maximum likelihood tree constructed from SNPs found in the coding regions of the genomes of ISS-F3, ISS-F4, and 8 other F. oxysporum strains (see Table S3 in Data Set S5 for reasons for inclusion in analysis). ISS-F3/F4 form their own clonal lineage distinct from Fo47 and the other strains. A similar tree was inferred when all SNPs across the genome were analyzed. (B) The frequency of SNPs across the entire genome (black), the noncoding region of the genome (purple), and the coding region of the genome (yellow) is summarized in the bar graph. Fol016 and 26381, well-defined strains, known to be different than Fo47, had a lower frequency of SNPs than ISS-F3/F4.

SNP genotyping was the second *in silico* comparative method used to determine the relatedness of the ISS isolates to that of Fo47. Using Fo47 as the reference genome, the number of SNPs in the genomes of ISS-F3/F4 compared to Fo47 was calculated, along with other strains as a comparison. These additional strains are listed in Table S3 in Data Set S5, along with the reasons for choosing them. Phylogenies of these isolates inferred using maximum likelihood from SNP sequences in coding regions across every genome indicate that they are distinct from Fo47 and the other strains included (Fig. 2A). Similar results were obtained with SNPs across the entire genome (coding and noncoding regions) (data not shown). A summary of the total number of SNPs across the genome, the number of SNPs in coding regions, and the number of SNPs in noncoding regions is displayed in Fig. 2B. The total numbers of SNPs in ISS-F3/F4 were 150,000 and 152,000, which are more than *lycopersici* Fol016 and *radicis-lycopersici* 26381 (also known as CL57), which are well-defined, characterized strains, known to be different than Fo47.

### Annotation of ISS-F3/F4 genes.

**FIG 3:**
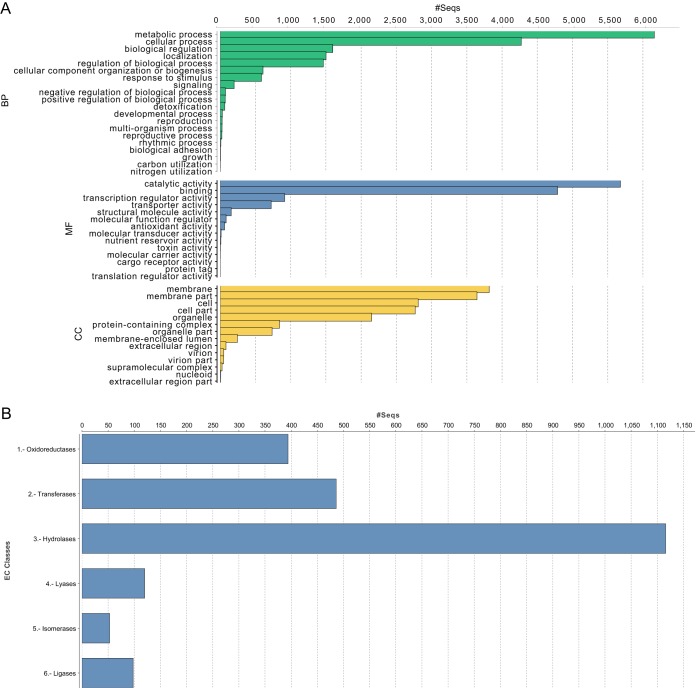
Summary of gene ontology (GO) annotation of the predicted genes in ISS-F3/F4. Predicted genes in ISS-F3/F4 were predicted using AUGUSTUS and then annotated using Blast2GO. Functions could be assigned to 10,789 of the 16,648 genes in ISS-F3 and 11,305 of the 16,729 genes in ISS-F4. (A) Bar charts summarizing the most abundant GO terms for ISS-F3. BP, biological processes; MF, metabolic function; CC, cellular components. (B) Bar charts summarizing the abundance of enzyme classes in ISS-F3. NB, ISS-F3/F4 had the same distributions of GO and enzyme class abundances, and thus, only ISS-F3 is shown for simplicity. The distributions in ISS-F3/F4 were similar to Fo47 (non-plant-pathogenic soil isolate) and FOSC-3a (clinical isolate) (data not shown).

Using the gene prediction program AUGUSTUS (30), ISS-F3 was predicted to contain 16,648 genes and ISS-F4 had 16,729 genes. This number is very close to the predicted number of genes (17,735) for the model strain, F. oxysporum f. sp. *lycopersici* 4287 (20, 31). The predicted genes were annotated with BLAST2GO, and 47% of the genes in ISS-F3 and 56% in ISS-F4 could be matched to known proteins while the remaining genes were listed as hypothetical (Data Sets S1 and S2). Among the predicted genes, 273 genes in ISS-F3 and 76 genes in ISS-F4 had no similarity to fungal genes in the NCBI database (Data Sets S1 and S2) and could possibly represent novel, unidentified fungal proteins. Gene Ontology (GO) functional annotation was also performed with BLAST2GO, and 65% of the predicted genes in ISS-F3 and 68% in ISS-F4 could be assigned a function (Data Sets S1 and S2). The most frequently represented GO functional assignments were metabolic and cellular processes (category: biological processes), catalytic activity and binding (category: metabolic function), and membrane and membrane part components (category: cellular component) (Fig. 3A). A summary of the 50 most abundant GO terms in each of the 3 categories mentioned above is presented in Fig. S1. Approximately 15% of the predicted genes were classified as enzymes, with hydrolases dominating followed by transferases (Fig. 3B).

10.1128/mSystems.00345-18.1 FIG S1 GO annotation of F. oxysporum ISS-F3/F4 strains. Bar graphs showing the 50 most abundant GO terms for ISS-F3 (A) and ISS-F4 (B) for each of the three categories: biological processes (BP), top; metabolic function (MF), middle; and cellular components (CC), bottom. Download FIG S1, PDF file, 0.1 MB. Copyright © 2019 Urbaniak et al. 2019 Urbaniak et al. This content is distributed under the terms of the Creative Commons Attribution 4.0 International license.

10.1128/mSystems.00345-18.6 DATA SET S1 Summary of BLAST results, gene ontology (GO), and InterProScan (IPS) annotations for ISS-F3. Download Data Set S1, XLSX file, 9.5 MB. Copyright © 2019 Urbaniak et al. 2019 Urbaniak et al. This content is distributed under the terms of the Creative Commons Attribution 4.0 International license.

10.1128/mSystems.00345-18.7 DATA SET S2 Summary of BLAST results, gene ontology (GO), and InterProScan (IPS) annotations for ISS-F4. Download Data Set S2, XLSX file, 9.6 MB. Copyright © 2019 Urbaniak et al. 2019 Urbaniak et al. This content is distributed under the terms of the Creative Commons Attribution 4.0 International license.

**FIG 4:**
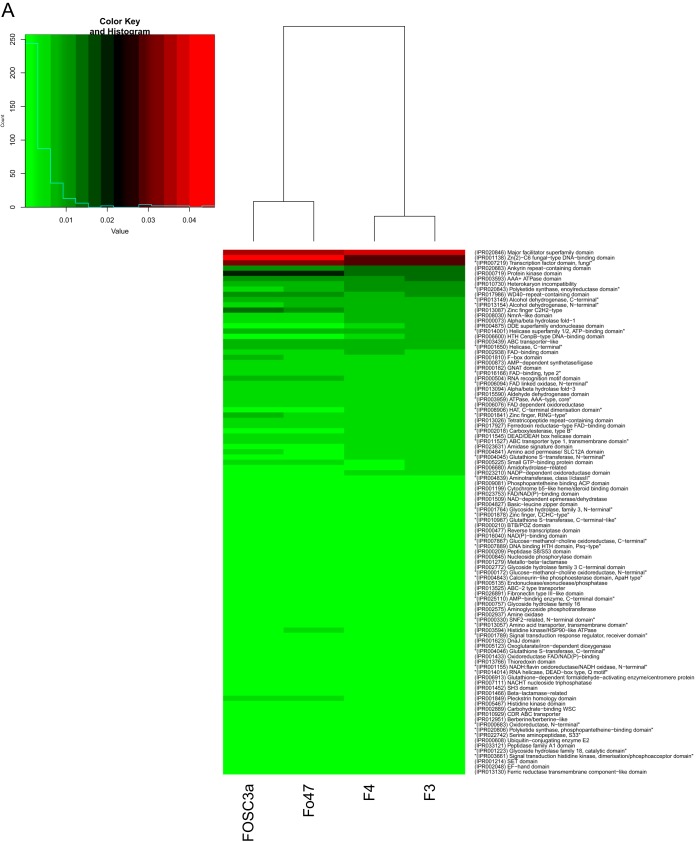

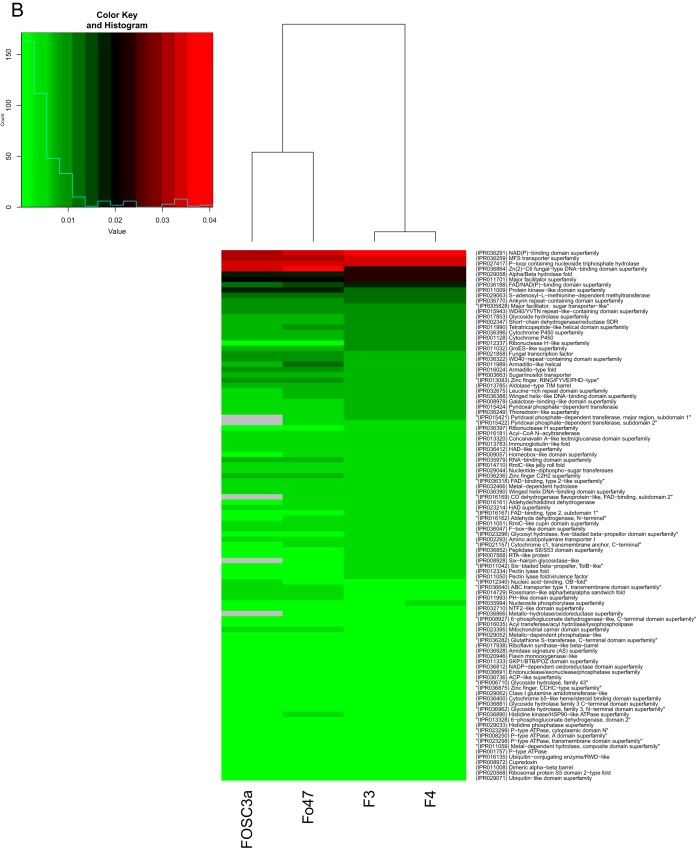
Annotation of protein domains and protein families with InterProScan. Protein families were classified and domains were predicted using InterProScan. The heat map shows the relative abundances of the top 100 most abundant domains (A) and families (B) in ISS-F3/F4, in addition to a non-plant-pathogenic soil isolate (Fo47) and a clinical isolate, isolated from a patient with fusariosis (FOSC-3a). Red represents high relative abundances, and green represents low relative abundances.

Protein families were classified and domains predicted with InterProScan, and 91% of the predicted proteins in ISS-F3/F4 could be assigned a function. Abundant protein domains in ISS-F3/F4 were the major facilitator superfamily (MFS) domain, the Zn(2)-C6 fungal-type DNA-binding domain, the fungal transcription factor domain, the ankyrin repeat-containing domain, and the protein kinase domain (Fig. 4A). The abundant protein families were the NAD(P)-binding domain superfamily, MFS transporter superfamily, P-loop-containing nucleoside triphosphate hydrolase, Zn(2)-C6 fungal-type DNA-binding domain superfamily, and the alpha/beta hydrolase fold (Fig. 4B). A comparison was performed between the ISS strains isolated during this study and a non-plant-pathogenic biocontrol soil isolate (Fo47) and a clinical isolate (FOSC-3a). While the most abundant domains and families in ISS-F3/F4 were also the most abundant ones in Fo47 and FOSC-3a, the collective proportions differed, with Fo47 and FOSC-3a more similar to each other than to ISS-F3/F4 (Fig. 4). There were also many domains and families that were present in Fo47 and FOSC-3a that were not found in the ISS-F3/F4 genomes and vice versa (Fig. S2). Data Set S3 shows the full list of protein domains and families present in each of the different strains and the relative abundances of each within each genome.

10.1128/mSystems.00345-18.2 FIG S2 Annotation of protein domains and protein families with InterProScan. Protein families were classified and domains predicted using InterProScan. Heat maps in panels A and B capture the presence/absence profiles of all 2,468 domains and 3,061 families detected in the 4 strains. Gray bars indicate that a specific domain (A) or family (B) was absent in a specific genome. Of the family and domains that are present, the color gradient shows the relative abundances within the genome, with light red being the most relatively abundant and light green being the least relatively abundant. Download FIG S2, PDF file, 0.2 MB. Copyright © 2019 Urbaniak et al. 2019 Urbaniak et al. This content is distributed under the terms of the Creative Commons Attribution 4.0 International license.

10.1128/mSystems.00345-18.8 DATA SET S3 Summary of relative abundances of protein families (A) and protein domains (B) in the genomes of four F. oxysporum strains. Download Data Set S3, XLSX file, 0.2 MB. Copyright © 2019 Urbaniak et al. 2019 Urbaniak et al. This content is distributed under the terms of the Creative Commons Attribution 4.0 International license.

10.1128/mSystems.00345-18.9 DATA SET S4 List of effector genes used in Fig. 1B. Download Data Set S4, XLSX file, 0.01 MB. Copyright © 2019 Urbaniak et al. 2019 Urbaniak et al. This content is distributed under the terms of the Creative Commons Attribution 4.0 International license.

10.1128/mSystems.00345-18.10 DATA SET S5 Table S1 is a summary of 65 genomes downloaded from GenBank that were used to generate *F. oxysporum* strain-specific k-mers (ftp://ftp.ncbi.nlm.nih.gov/genomes/genbank/fungi/Fusarium_oxysporum/). Table S3 is a list of F. oxysporum strains in addition to ISS-F3 and ISS-F4 that were used in SNP analysis. The “Reason” column lists the reasons for including these strains for comparison. Download Data Set S5, XLSX file, 0.01 MB. Copyright © 2019 Urbaniak et al. 2019 Urbaniak et al. This content is distributed under the terms of the Creative Commons Attribution 4.0 International license.

### Identification of candidate pathogenicity proteins and secondary metabolites.

Polyketide synthases (PKSs) are large, multidomain protein complexes that catalyze the sequential production of a diverse array of secondary metabolites (SM) with a range of biological activities, including pigmentation, plant growth regulation, plant pathogenicity/invasion, and toxicity toward humans and animals, with some having potential for drug development and human benefit (32–34). Different PKSs catalyze the synthesis of structurally distinct polyketides, and to date 52 PKSs have been identified in *Fusarium* with 16 found in F. oxysporum (35).

Due to the importance of PKS in SM production, predicted proteins composed of PKS domains, based on the InterProScan results above, were further studied to identify which PKSs (and the resulting polyketides) ISS-F3/F4 have the potential to express. The amino acid sequences of ISS-F3/F4 proteins with PKS domains were compared using BLAST against a database composed of PKSs identified from 12 strains of F. oxysporum and 9 other *Fusarium* species (35).

**FIG 5:**
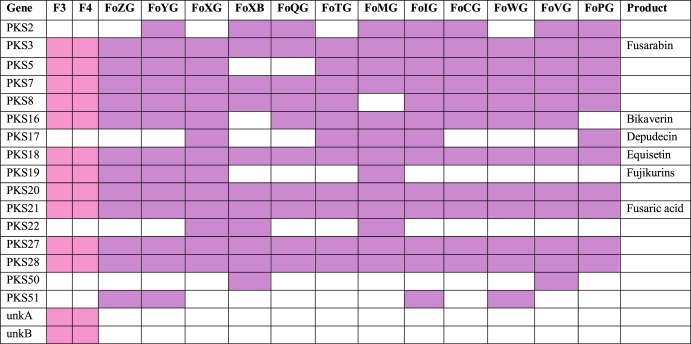
Presence/absence profile of polyketide synthases (PKSs) detected in the genomes of ISS-F3/F4. The sequences of predicted genes that had a ketoacyl synthase domain (one of 3 essential domains in polyketide synthases [PKSs]) were compared with BLAST against a PKS database (35) to determine which PKSs ISS-F3/F4 had the ability to produce. Eleven PKSs found in ISS-F3/F4 are also present in 12 other F. oxysporum species that have been previously studied (35). However, there were two additional PKSs, unkA and unkB, in ISS-F3/F4 that did not have any matches in the database. The first column represents the PKS, the last column shows the polyketide that the PKS makes (if known), and the middle columns indicate the F. oxysporum strains. Colored boxes indicate the presence of a specific PKS in the genome. FoZG, Fo47; FoYG, FOSC-3a; FoXG, *lycopersici* 4287; FoXB, Fo5176; FoQG, *raphani* NRRL 54005; FoTG, *vasinfectum* NRRL 25433; FoMG, *melonis* NRRL 26406; FoIG, *cubense* tropical race 4; FoCG, *radicis-lycopersici* 26381; FoWG, MN25; FoVG, *pisi* (HDV 247); FoPG, *conglutinans* race 2 NRRL 54008. Amino acid sequences for the unknown PKSs in ISS-F3 can be found in Data Set S1 (g14942 and g11694), and those for ISS-F4 can be found in Data Set S2 (g15849 and g12099). NB, the sequences are identical between ISS-F3/F4.

The BLAST searches revealed the presence of 11 PKSs in the genomes of ISS-F3/F4 which have all been detected in other F. oxysporum strains (Fig. 5). Compared to the 12 F. oxysporum strains, the presence/absence PKS profile of ISS-F3/F4 best resembled that of Fo47, followed by FOSC-3a (Fig. 5). In addition to the 11 PKS proteins identified in ISS-F3/F4, there were two proteins with ketoacyl synthase domains that did not match anything in the database and could possibly represent novel, yet-unidentified *Fusarium* PKSs.

A more extensive SM assessment was performed with antiSMASH, which identified 47 biosynthetic gene clusters in the genome of ISS-F3 and 46 in ISS-F4. To put this finding into perspective, 44 biosynthetic gene clusters were identified in Fo47 while 47 clusters were present in the FOSC-3a. All four strains had biosynthetic gene clusters in their genomes that are involved in the production of type 1 polyketides, nonribosomal peptides, indoles, terpenes, and polyketide/nonribosomal peptide hybrids. While ISS-F3, ISS-F4, and Fo47 had clusters involved in type III polyketide production, FOSC-3a did not appear to have that machinery in its genome. A summary of the biosynthetic gene clusters detected in the ISS-F3, ISS-F4, Fo47, and FOSC-3a genomes is shown in Fig. S3.

10.1128/mSystems.00345-18.3 FIG S3 A comparison of biosynthetic gene clusters in F. oxysporum strains. Biosynthetic gene clusters, and thus the ability to produce secondary metabolites, were analyzed in ISS-F3/F4 using antiSMASH. Included for comparison were the biosynthetic gene clusters in Fo47 (a non-plant-pathogenic soil isolate) and FOSC-3a (a clinical isolate). The boxes with numbers listed to the right of the strain name indicate the number of clusters in the genome for that specific type (i.e., for T1pks there were 7 clusters in strain ISS-F3). The letters indicate the name of the compound produced or the name of the polyketide synthase (PKS) or nonribosomal peptide synthase (NRPS). Download FIG S3, PDF file, 0.2 MB. Copyright © 2019 Urbaniak et al. 2019 Urbaniak et al. This content is distributed under the terms of the Creative Commons Attribution 4.0 International license.

### Virulence of F. oxysporum strains estimated in C. elegans model.

F. oxysporum f. sp. *lycopersici* strain 4287, a well-characterized model pathogen of tomato plants, was shown to produce systemic disease in immunocompromised mice, resulting in a high death rate (36). Utilizing F. oxysporum strain 4287 knockout mutants that exhibit altered virulence in tomato plants, it was shown that certain virulence factors essential in plant pathogenesis were dispensable in mammalian pathogenesis and vice versa (36). This raises the possibility that certain phytopathogens could also be virulent in humans depending on their health status. This is concerning given the fact that a recent study on the impact of space on fungal pathogenesis has shown that Aspergillus fumigatus isolates cultured from ISS surfaces were more virulent in a zebrafish model of invasive aspergillosis than Earth-based clinical isolates (37).

This prompted the examination of the virulence potential of ISS-F3/F4 in an immunocompromised (MAPKK-deficient) C. elegans model of invasive fusariosis. Included for comparison was an *F. oxysporum* strain, IMV00293, isolated in the aftermath of the Chernobyl disaster, flown to the ISS and grown for 12 days (“293gr”), and grown concomitantly on Earth (“293sp”).

**FIG 6:**
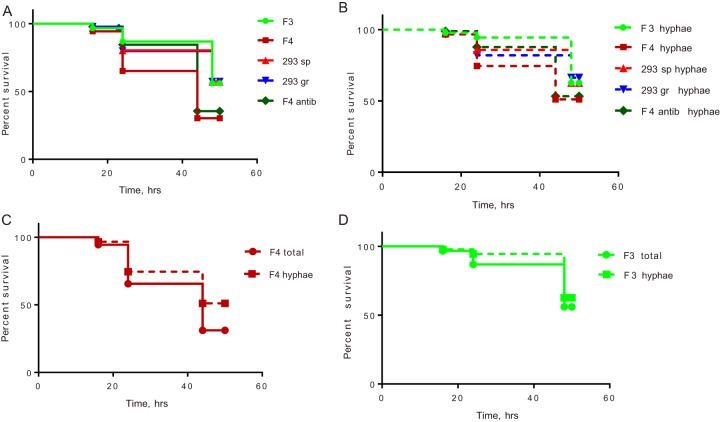
Virulence of Fusarium oxysporum strains in C. elegans AU37 model. (A) Kaplan-Meier survival curves to determine total death of worms caused by F. oxysporum strains (*n* = 90 worms per fungal strain, results accumulated from 3 different experiments, 3 biological replicates per experiment, 10 worms per replicate). *P* < 0.0001 for F4 and F4antib compared to all other strains. (B) Kaplan-Meier survival curves to determine the hypha-related death of worms (*n* = 90 worms per fungal strain, results accumulated from 3 different experiments, 3 biological replicates per experiment, 10 worms per replicate). *P* = 0.0129 for F4 and F4antib compared to all other strains. (C and D) Comparison of hypha-related and total deaths of C. elegans caused by ISS-F4 (C) and ISS-F3 (D). Total deaths of C. elegans caused by ISS-F4 were significantly higher than the deaths associated with hyphae piercing through the body (*P* = 0.0102, *n* = 90, log rank [Mantel-Cox] test). There is no statistical difference between total and hypha-related deaths caused by ISS-F3 (*P* = 0.2465, *n* = 90). NB, 293sp is the IMV00293 strain that was isolated from Chernobyl and grown on the ISS for 12 days, and 293gr is IMV00293 that was concomitantly grown for 12 days on Earth.

**FIG 7:**
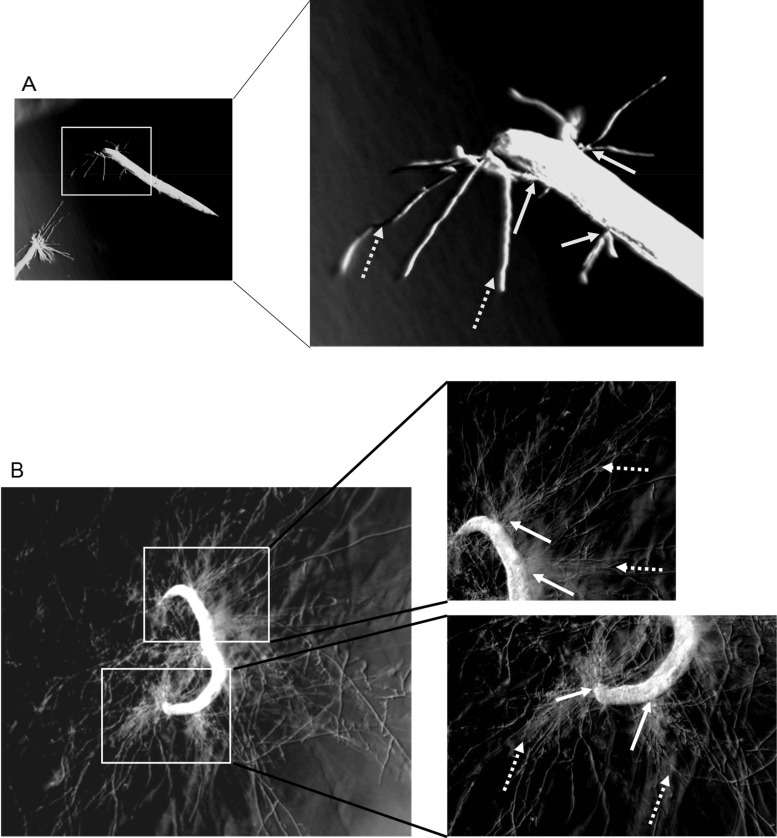
Images of C. elegans AU37 with hyphae of F. oxysporum ISS-F4 piercing through worm body. Microscopic images taken at 22 h (A) and 46 h (B) after coincubation of C. elegans with ISS-F4 conidia. Solid arrows point out the hyphae piercing through the worm body. Dotted arrows show growing extended hyphae that initially penetrated from the intestinal tube. The images were taken with the Olympus SZX16 microscope (×5 magnification).

Figure 6 demonstrates the effect of five strains of F. oxysporum on mortality of C. elegans AU37. Kaplan-Meier survival curves of C. elegans analyzed with the log rank (Mantel-Cox) test demonstrated that the virulence of F. oxysporum ISS-F4 was significantly greater than other strains (*P* < 0.0001) (Fig. 6A). The culturing conditions for fungal growth such as growing without antibiotic or with antibiotic used to prepare conidia did not affect ISS-F4 killing ability (*P* = 0.6888). The majority of worms died due to F. oxysporum hyphae piercing through the worm body (Fig. 6B); however, ISS-F4 virulence was attributed to both hypha- and non-hypha-related killing (Fig. 6C), whereas ISS-F3 virulence was attributed solely to hypha-related mortality (Fig. 6D). The hypha-related killing of ISS-F4 was still significantly greater than other strains (*P* = 0.0129) (Fig. 6B). Importantly, while ISS-F3, 293gr, and 293sp had less killing capability than ISS-F4, they should be still considered virulent, as their related mortality for worms reached 50% at 50 h. To note, there were no differences in mortality between 293gr and 293sp, indicating that growth on the ISS (12 days) did not change the virulence of this strain. Figure 7 shows microscopic images of C. elegans worms with hypha penetration, 22 h and 46 h after exposure to ISS-F4 conidia.

## DISCUSSION

This study presents the genomic analysis of two F. oxysporum isolates, cultured from the ISS, during an ongoing microbial tracking study. The phylogenetic placement of these two strains based on EF-1α nucleotide sequence and whole-genome SNPs shows that they are within the genetic variation of terrestrial strains and that their ability to thrive in the space environment was not contingent on enhanced genetic variation.

F. oxysporum strains are well known for their plant pathogenicity, and for successful infection of its plant host, effector proteins are secreted from the fungus that aid in the colonization process. Performing disease assays to determine host specificity is both laborious and time-consuming; however, a recent molecular screening method based on 104 effector proteins was developed by van Dam et al. which can predict host specificity in F. oxysporum (24). The presence/absence of these effector proteins in ISS-F3/F4 was determined and compared to 62 other strains from a variety of *formae speciales*. The ISS strains contained relatively few candidate effector genes compared to phytopathogens, with numbers similar to that detected in two non-plant-pathogenic strains, Fo47 and MN14. The ISS isolates also did not contain any of the 14 secreted in xylem (SIX) effector genes, which encode cysteine-rich secreted proteins that appear to manipulate host defenses to promote infection (38). Based on the effector content, it is unlikely that ISS-F3/F4 are plant pathogens. VEG-01C1 and VEG-01C2, isolated from *Z. hybrida* plants grown on the ISS and whole-genome sequences (39) also displayed relatively few effector genes (Fig. 1B) and were unable to infect healthy *Z. hybrida* plants (G. Massa, personal communication).

Based on effector content and EF-1α phylogeny, ISS-F3/F4 appeared to be the most closely related to Fo47, a non-plant-pathogenic soil isolate known for its biocontrol properties. Based on the number of SNPs across the genome, the phylogeny inferred using maximum likelihood from SNP sequences in both coding and noncoding regions, and the k-mer analysis, ISS-F3/F4 were not Fo47 or identical to any of the 65 strains compared in this study. Thus, it appears that these ISS isolates represent a genomically unique strain, which has not yet been identified and characterized to date.

The ISS is a hermetically sealed system, closed off from its surrounding environment, with microbial introduction being of terrestrial origin from astronauts and payloads. ISS-F3/F4 were most likely introduced in the same fashion, so it begs the question why they had been identified only on the ISS and not on Earth, where 150 *formae speciales* have already been characterized (2). One hypothesis is that ISS-F3/F4 are present in relatively low abundances on Earth but were selected because of their ability to withstand ISS-associated environmental pressures such as increased radiation, which compared to terrestrial indoor buildings, is 100 times greater. In a recent study by Byrne et al., it was found that Escherichia coli developed extreme resistance to ionizing radiation, comparable to that observed with Deinococcus radiodurans, with mutations in only 9 genes, all of which happen to be involved in radiation resistance (40). When these mutations were repaired, resistance was lost and the cells became sensitive to radiation (40). This paper showed for the first time that evolution of resistance may occur via genetic adaptation rather than passive mechanisms based on changes in cellular proteins. We therefore hypothesized that based on the sequences of five genes known to have a role in radiation resistance in fungi, ISS-F3/F4 would appear to be more closely related to IMV00293 (Chernobyl isolate) and VEG-01C1/VEG-01C2 (ISS *Z. hybrida* isolates) (as they are strains also isolated from high-radiation environments) compared to 64 other F. oxysporum strains. An inferred maximum likelihood tree was constructed from concatenated sequences of *rad54*, *rad53*, *rad51*, *rad2*, and *phr1* (a photolyase) found in ISS-F3, ISS-F4, IMV00293, VEG-01C1, VEG-01C2, and 64 other F. oxysporum strains. The branching observed with the radiation resistance genes was similar to that observed with EF-1α in which ISS-F3/F4 grouped together, apart from IMV00293, VEG-01C1, and VEF-01C2, which grouped together, with all five isolates being within the genetic variation of the 64 other strains included (see Fig. S4 in the supplemental material). A proteomics approach may provide more insight into whether ISS-F3/F4 have a selective advantage for survival on the ISS and what that may entail.

10.1128/mSystems.00345-18.4 FIG S4 Sequence comparison of radiation resistance genes among 69 F. oxysporum strains. An inferred maximum likelihood tree was constructed from concatenated sequences of *rad54*, *rad53*, *rad51*, *rad2*, and *phr1* (a photolyase), genes that have been shown to play a role in radiation resistance in fungi. Fusarium proliferatum and *Fusarium fujikuroi* were used to root the tree. Since ISS-F3, ISS-F4, IMV00293, VEG-01C1, and VEG-01C2 were all cultured from radiation-rich environments, it was hypothesized that they would share similar sequences in these genes, distinct from the other 65 F. oxysporum strains. However, as the tree shows, this was not the case. Download FIG S4, PDF file, 0.05 MB. Copyright © 2019 Urbaniak et al. 2019 Urbaniak et al. This content is distributed under the terms of the Creative Commons Attribution 4.0 International license.

The genomes of ISS-F3/F4 were largely made up of gene ontology categories involved in metabolic and cellular processes and catalytic activity and binding. Overall, the relative abundances of GO categories for ISS-F3/F4 were similar to that observed in FOSC-3a, a clinical isolate, and Fo47, a biocontrol, non-plant-pathogenic strain. However, there were differences in the relative abundances and presence/absence of protein domains and families between ISS-F3/F4 and FOSC-3a and Fo47, highlighting the uniqueness of these strains. One such difference was the higher relative abundance of genes encoding polyketide synthase domains in ISS-F3/F4. Each PKS makes a unique polyketide, which collectively represent structurally diverse natural products with commercial significance for the pharmaceutical industries (41). A testament to its importance is reflected in the annual sales of pharmaceuticals derived from polyketides, which routinely reach $20 billion (42). For example, the anticancer drugs tetracycline and doxorubicin, the cholesterol-lowering drug lovastatin, the antibiotics erythromycin and rifamycin, and the fungicide strobilurin are all polyketides that have been isolated from either bacteria or fungi (43). A BLAST search against a PKS database (35) showed that ISS-F3/F4 had the genomic machinery to produce 13 polyketides, 11 of which have been found in other *F. oxysporum* strains. The two sequences in ISS-F3/F4 that did not have any matches in the PKS database did match F. oxysporum hypothetical proteins in NCBI. A more extensive analysis using proteomics and metabolomics will be performed to elucidate the natural product repertoire of ISS-F3/F4.

F. oxysporum, though a well-known plant pathogen, may cause various infections in humans and is a cause of emerging infections in immunodeficient patients (44–46). Vertebrate and nonvertebrate models are well established to test pathogens for their potential virulence (36, 47–49). For virulence testing of F. oxysporum, a host is usually immunosuppressed to make it susceptible to fungal infection (50). Symptoms of disseminated fusariosis caused by F. oxysporum are remarkably similar between animals and humans. They include development of invasive structures, including hypha microconidia and chlamydospores, causing massive colonization of organs, and which can cause thrombosis and necrosis (4, 50, 51). The nonvertebrate host C. elegans, a free-living soil nematode, is one of the most useful models to study the virulence of fungi and is well established by many researchers (52–54). C. elegans has an innate immune response mechanism that is activated in the response to pathogens, including fungi (55, 56). A key component of the C. elegans immune response is the p38 mitogen-activated protein kinase (MAPK) pathway (57). SEK-1 encodes a mitogen-activated protein kinase kinase (MAPKK) in this pathway and thus plays a vital role in pathogen resistance in C. elegans (58). For this reason, SEK-1 mutants are considered “immunocompromised” worms. Matricidal hatching of progeny inside the parent worms occurs in SEK-1-deficient C. elegans, and therefore, a mutation in *glp-4*, a locus required for normal proliferation of the germ line (59), is required to avoid bias effects due to progeny hatching. The *glp-4* mutants are sterile at the restrictive temperature of 25°C, at which C. elegans experiments are performed.

Using the *glp-4/sek-1* mutant strain AU37 of C. elegans obtained from the Caenorhabditis Genetics Center, we observed that ISS-F4 was the most virulent, even compared to ISS-F3, despite the fact that ISS-F3/F4 have very similar genomes and initially were thought to be clonal derivatives of each other. Since ISS-F4 demonstrated killing ability by both hypha piercing-related and -unrelated mechanisms, it is likely that it produces secreted virulence factors. It is known that secreted proteins such as RP-1-like (60) and secreted in xylem (SIX) proteins (61), which are regulated by *Fusarium* transcription factor 1 (FTF1) (62), are required for full virulence of F. oxysporum. Further work is required to determine if there are differences in production and regulation of the secreted factors between ISS-F3/F4 strains and if time-dependent exposure to the ISS environment may affect this regulation.

## MATERIALS AND METHODS

### Isolation of fungal strains.

Surfaces (1 m^2^) were sampled on the ISS using premoistened 9-in. by 9-in. polyester wipes (ITW Texwipe, Mahwah, NJ) that were prepared as part of an ongoing microbial tracking study during flight sampling 3 (6 May 2016). Wipes were stored at room temperature (RT) before being brought back to Earth, 6 days after sampling, where they were processed as described previously (63). Material from the wipes was plated, 100 μl, on potato dextrose agar (PDA) supplemented with chloramphenicol (final concentration, 100 μg/ml) and incubated at 25°C for 7 days. Fusarium oxysporum, identified by morphology and ITS region sequencing, was isolated from the ISS dining table. The two isolates were designated ISS-F3 and ISS-F4.

### Whole-genome sequencing. (i) Genomic DNA extraction.

Freezer stocks of ISS-F3/F4 were plated on potato dextrose agar (PDA) plates and incubated at 25°C for 5 days, at which time the entire plate was covered with fungal growth. The growth over the entire plate was scraped into a sterile mortar and pestle containing a small amount of liquid nitrogen. The colonies were ground into a powder and then transferred into PowerBead tubes from the MoBio PowerSoil DNA isolation kit (Qiagen, USA; catalog number 12888), and the extraction protocol was followed per the manufacturer’s instructions, except for the last step, in which DNA was eluted in 80 μl of solution C6 (i.e., elution buffer). DNA was stored at −20°C until sent for sequencing.

### (ii) Sequencing and assembly.

Genomic DNA was paired-end sequenced (2 × 100 bp) with a 350-bp insert size on the Illumina HiSeq 2500 platform by Macrogen (Rockville, MD, USA). Totals of 48 million reads for F3 and 42 million reads for F4 were generated. Trimmomatic on the Galaxy server (https://usegalaxy.org) was used to remove the sequencing adaptors (settings: maximum mismatch, 2; accuracy of the match between the two adaptor-ligated reads, 30; accuracy of the match between any adaptor, 10) and to trim the leading and trailing ends (settings: minimum quality required to keep a base, 3). Postprocessed reads were *de novo* assembled using ABySS version 2.0.2 (19) using the default settings. Different k-mer sizes ranging from 68 to 90 were tested in order to find the one that gave the highest *N*_50_ score and lowest number of scaffolds/contigs, after assembly. The k-mer size 86 gave the best results for both F3 and F4, and the resulting assemblies were used for downstream analyses.

### Phylogenetic analysis.

The phylogeny of the newly identified strains was assessed by extracting the full-length gene sequence of translation elongation factor 1 alpha (EF-1α) from each of the genomes using BLASTN. The sequences were aligned using MUSCLE with default parameters (64), and phylogeny was inferred with PhyML (65) (1,000 bootstraps; percent bootstrap confidence is shown in red numbers in the tree). The trees were visualized using ETE 3 (66). The effector clustering was done on only the F. oxysporum strains, using the list of 104 curated F. oxysporum effector candidate genes and the same clustering method as described in the work of van Dam et al. (24). Briefly, 2.5-kb regions up- and downstream of a *miniature impala* (*mimp*) transposable element were scanned for potential open reading frames (ORFs) containing a signal peptide. This procedure was previously done by van Dam et al. (24) for 59 individual isolates of F. oxysporum, and the resulting effector candidate genes were grouped and merged into a list. Obvious false positives and transposable elements were removed, and multiple instances of the same effector were reduced to one representative. This curated list represents the “effectorome” of a varied set of plant-pathogenic *formae speciales* of F. oxysporum. Screening for the presence of the putative 104 effectors was done using BLASTN (as described above). A binary data matrix was generated containing presence (“1”) or absence (“0”’) of each candidate in each genome. This table was used as input for hierarchical clustering performed in R, using a Jaccard binary distance matrix and average linkage. The resulting matrix was visualized using the “heatmap3” package in R.

The sequences of the 10 phylogenetically informative loci used to compare ISS-F3/F4 were extracted from the genomes using the sequences of these genes in the reference genome of F. oxysporum f. sp. *lycopersici* 4287. The sequences were kindly provided by Li-Jun Ma from the University of Massachusetts at Amherst. The loci and chromosomal locations from the reference genome are as follows: chromosome 1, DNA-directed RNA polymerase III subunit RPC2 and RPB1; chromosome 4, minichromosome maintenance protein 3 and anaphase-promoting complex subunit 1; chromosome 5, two hypothetical proteins, FOXG 1751.3 and 2073.3; chromosome 7, DNA-directed RNA polymerase II subunit RPB2; chromosome 8, hypothetical protein FOXG 3560.3; and chromosome 9, clathrin heavy chain and DNA polymerase gamma (28).

### k-mer analysis for strain identification.

The KMC2 k-mer counter (29) tool was used to generate 50-bp-long k-mers from 65 F. oxysporum genomes that were downloaded from GenBank (ftp://ftp.ncbi.nlm.nih.gov/genomes/genbank/fungi/Fusarium_oxysporum/) (see Table S1 in Data Set S5 in the supplemental material). The KMC2 program was also used to determine which of the k-mers generated from each genome were unique to that genome. A subset of the unique k-mers were chosen, based on number of occurrences (chosen if they appeared once, instead of multiple times within the genome) and the type of sequence (the sequence did not contain a lot of repeats and had a good balance of GC and AT ratios). Using command line BLAST (version ncbi-blast-2.4.0+), the genomes of F3 and F4 were used as the query against the “database” of unique k-mer sequences. For ISS- F3 or ISS-F4 to be considered one of the 65 F. oxysporum strains, every k-mer in the database belonging to a specific strain had to have a perfect match against a sequence in either the F3 or F4 genome.

### Single nucleotide polymorphism detection and phylogenetic analysis.

We applied a reference-based analysis method to determine the relatedness of F3 and F4 to the reference genome, Fo47, and to 5 other F. oxysporum isolates. The list of these strains and the reasons for including them in the analysis are presented in Table S3 in Data Set S5. These publicly available genomes were downloaded from the NCBI sequence read archive (SRA). SNP identification was carried out using PhaME (version 1.0.2) (67), which uses Nucmer for pairwise alignment of genomes. Evolutionary analyses and phylogenetic trees were constructed as part of the PhaME pipeline. The numbers of SNPs spanning the entire genome, in coding regions, and in noncoding regions were extracted from the PhaME output using a custom Perl script and plotted in Prism (version 7). In order to detect SNPs in the coding regions, a gff annotation file of the reference genome (Fo47) was supplied as input.

### Gene prediction and annotation.

The genes of the assembled ISS-F3/F4 genomes were predicted with the AUGUSTUS gene prediction program using default settings and trained against Fusarium graminearum (30, 68). The amino acid sequences of the predicted genes were uploaded into Blast2GO (https://www.blast2go.com) (69), and annotation was performed with blastp-fast against the NCBI nonredundant database, set to the “fungi” taxonomy filter. The E value cutoff was set to <1.0E−10. Gene ontology classification and InterProScan were also performed with Blast2GO using the default settings.

### Identification of polyketides and secondary metabolites.

Predicted proteins that were annotated as polyketide synthases were further evaluated with the polyketide synthase database (35), which consists of amino acid sequences of PKSs from 12 F. oxysporum strains and 8 other *Fusarium* species. A blastp search was performed with F3 and F4 sequences, and a positive match was based on >95% identity and an E value of <1E−10.

Biosynthetic gene clusters were predicted with the fungal version of antiSMASH (https://fungismash.secondarymetabolites.org). The input files were the (i) assembled whole-genome nucleotide sequences and (ii) gff annotation file produced from AUGUSTUS. Default settings were used in the run.

### C. elegans model for Fusarium oxysporum virulence.

The *glp-4/sek-1* mutant strain AU37 of C. elegans obtained from the Caenorhabditis Genetics Center (http://www.cbs.umn.edu/research/resources/cgc) was used in the experiments.

C. elegans AU37 nematodes were synchronized for the experiment as previously described (70). Synchronized nematodes were then grown on E. coli OP50 lawns until they reached the L3-L4 stage. Then nematodes (about 300 worms) were collected and washed 3 times in 10 ml of M9 buffer (71) containing 50 µg/ml kanamycin. On the last washing, worms were incubated in 10 ml M9 buffer containing 50 µg/ml kanamycin for 1 h to clean the intestinal tube and surface of nematodes from E. coli. After cleaning, nematodes were poured on plain agar plates (1.5% agar in water) to soak the kanamycin solution into agar. At this point, synchronized fasting nematodes were ready for transferring into experimental fungal conidium suspensions.

Fungal conidium suspensions were prepared by growing the fungal strains on PDA plates and incubating them at 25°C for 7 days, at which time the plate was overgrown with growth. Eight milliliters of ST solution (8.5 g NaCl, 1 ml Tween 80 in 1 liter of water) was poured onto the plates, and a cotton swab was used to scrape all the moistened fungi off the plate. The liquid suspension on the plate was then transferred to a 50-ml Falcon tube, spun down at 3,500 × *g* for 5 min, and washed 3 times with sterile PBS. The pellet was then resuspended in 5 ml of sterile PBS. To count the number of conidia in the suspension, a 1/10 dilution was prepared using ST solution and conidia were counted using a hemocytometer under the 40× objective of a light microscope. Conidial stocks were diluted in PBS to 10^7^ conidia/ml prior to the experiment.

Twenty microliters of 10^7^-conidia/ml F. oxysporum spores were suspended in 1 ml of 30% (vol/vol) brain heart infusion broth (BHI broth; Remel, USA) prepared by dilution of BHI broth in M9 buffer. For each conidium suspension, 60 worms were transferred from plain agar plates into 1 ml of a conidium suspension and incubated for 1 h at 25°C without agitation. After incubation, the entire reaction volume of conidium suspension with worms was transferred to a 60-mm dish containing 2 ml of M9 buffer with 50 µg/ml kanamycin. Then, worms were collected by a micropipette and transferred to a 60-mm dish containing 3 ml of M9 buffer with 50 µg/ml kanamycin. This procedure was repeated 3 times using 3 ml of fresh M9 buffer with 50 µg/ml kanamycin in a 60-mm dish at each transfer. After final washing, 10 worms were transferred into each experimental 30-mm dish (3 dishes per one fungal strain conidium suspension) containing 2 ml of 30% BHI broth and 50 µg/ml kanamycin. Worms were incubated at 25°C without agitation, and worm death was estimated at 17, 24, 44, and 50 h after conidium exposure using an Olympus SZX16 microscope (×5 magnification) with a camera (Olympus DP30BW) for imaging.

### Data availability.

This Whole Genome Shotgun project has been deposited at DDBJ/ENA/GenBank under the accession numbers QUWZ00000000 (ISS-F3) and QUXA00000000 (ISS-F4). The version described in this paper is the first version.
