# Supplementary figures and images for: Genomic Characterization and Virulence Potential of Two Fusarium oxysporum Isolates Cultured from the International Space Station
Source: mSystems. 2019 Mar 19;4(2):e00345-18. doi: 10.1128/mSystems.00345-18 (PMC6426649; doi:10.1128/mSystems.00345-18)

F3

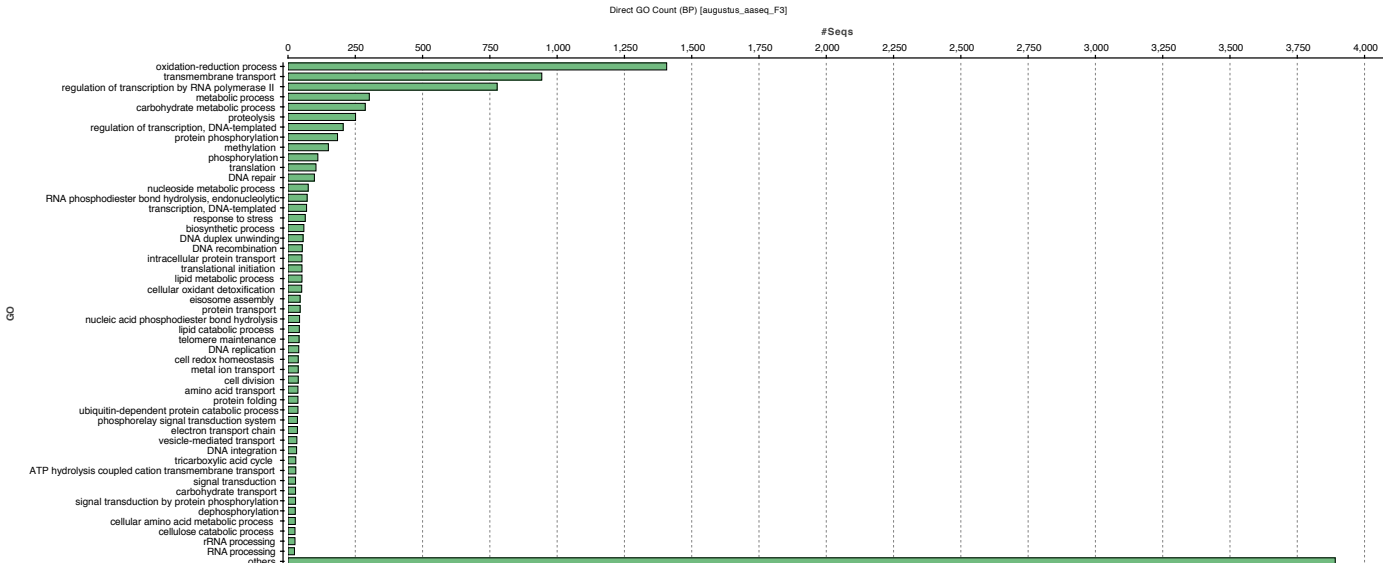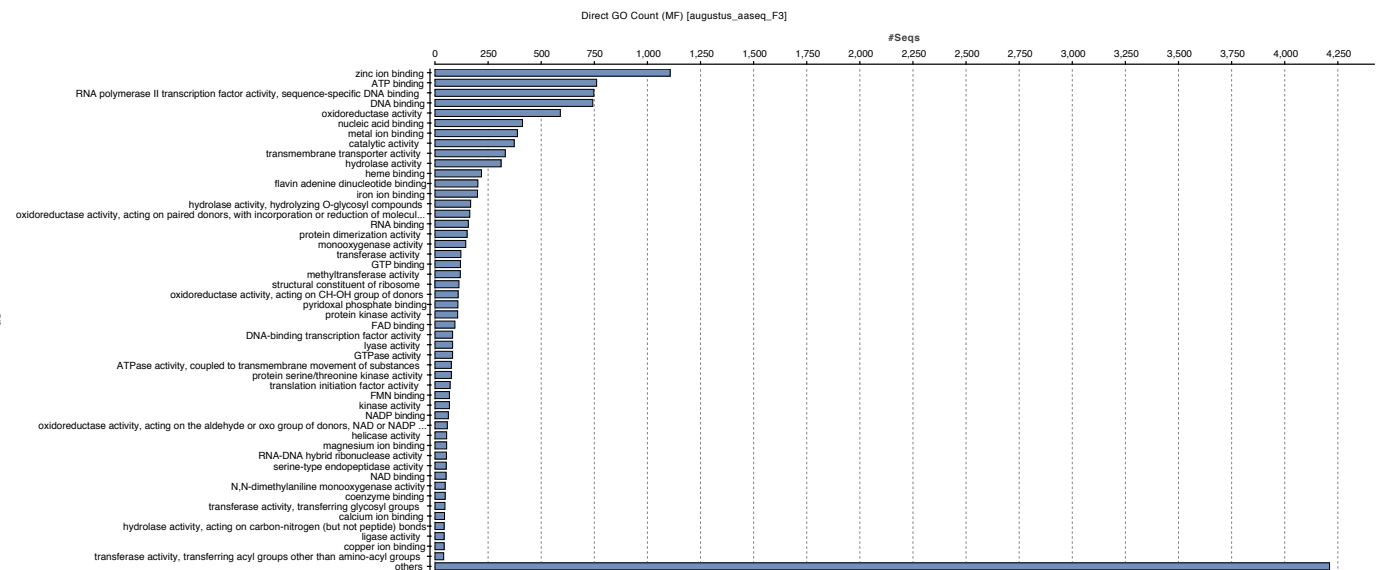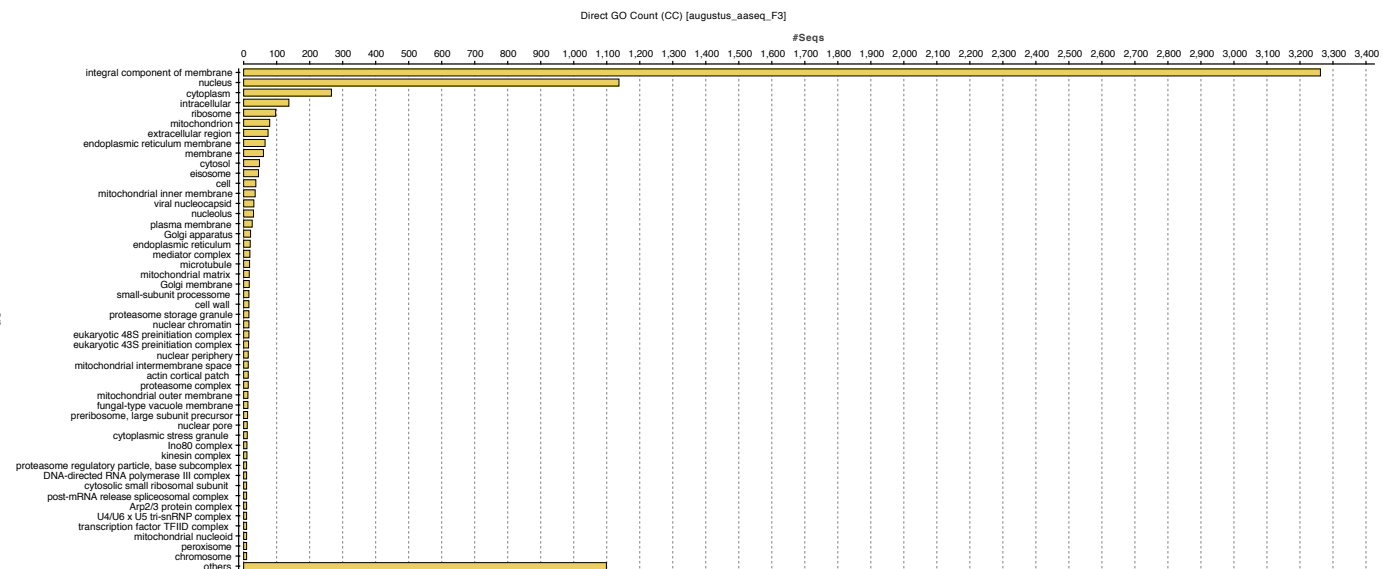

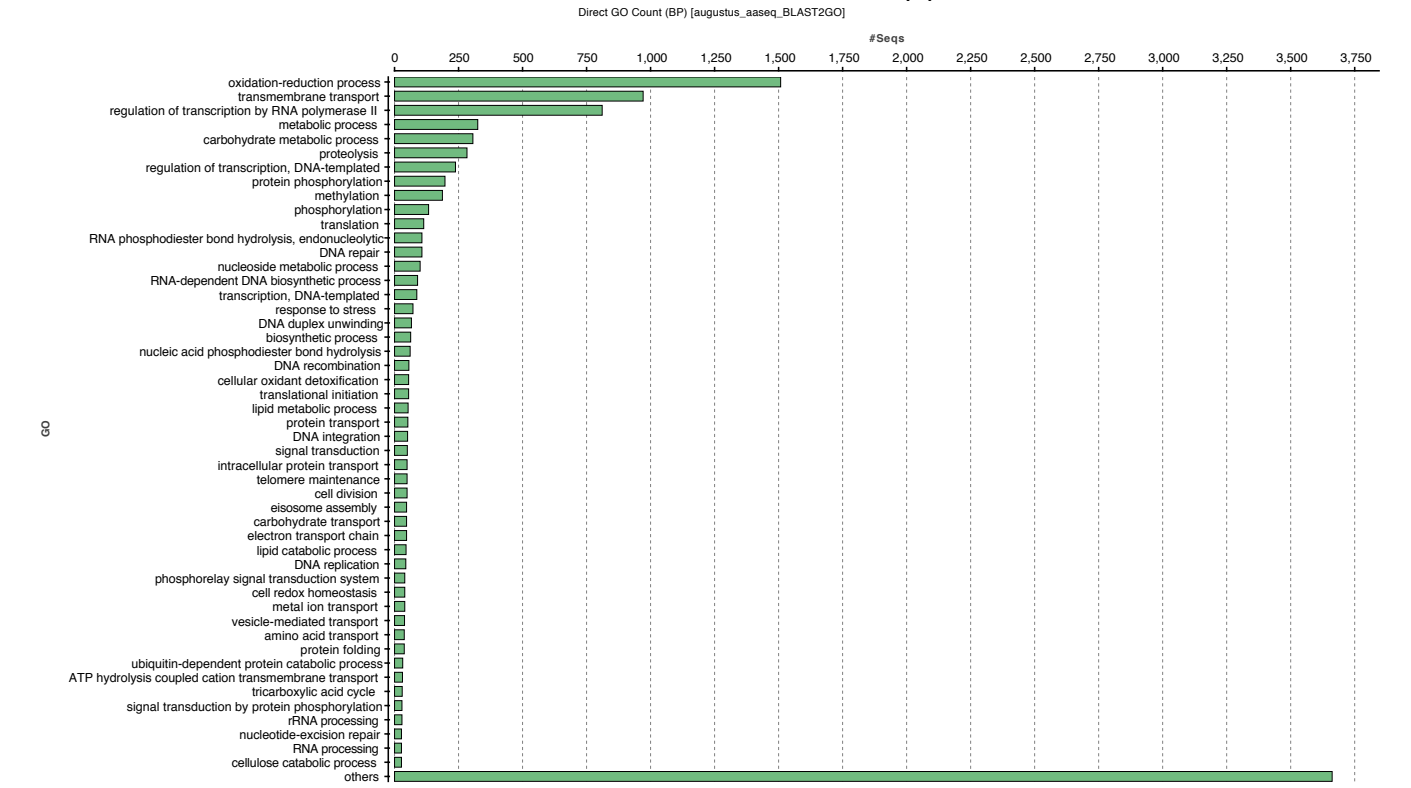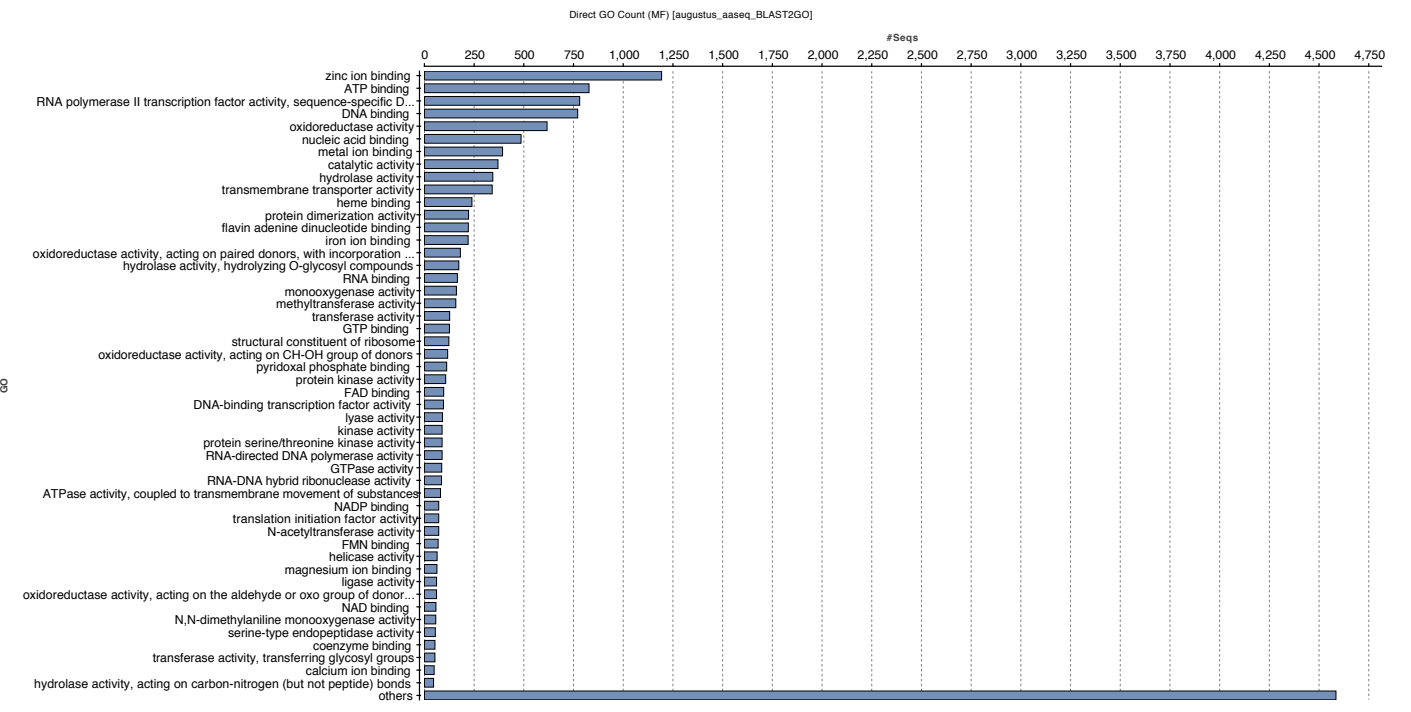

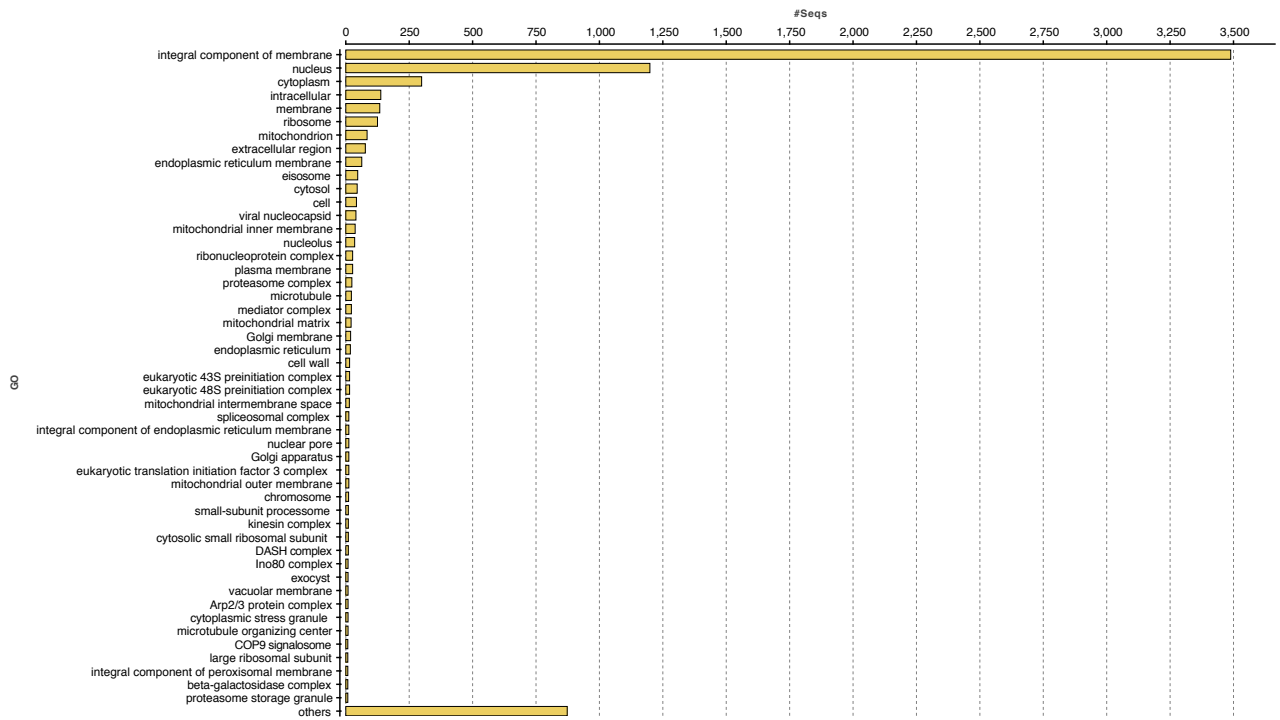

Supplement: FIG S1 [file mSystems.00345-18-sf001.pdf]

**Fig S3**

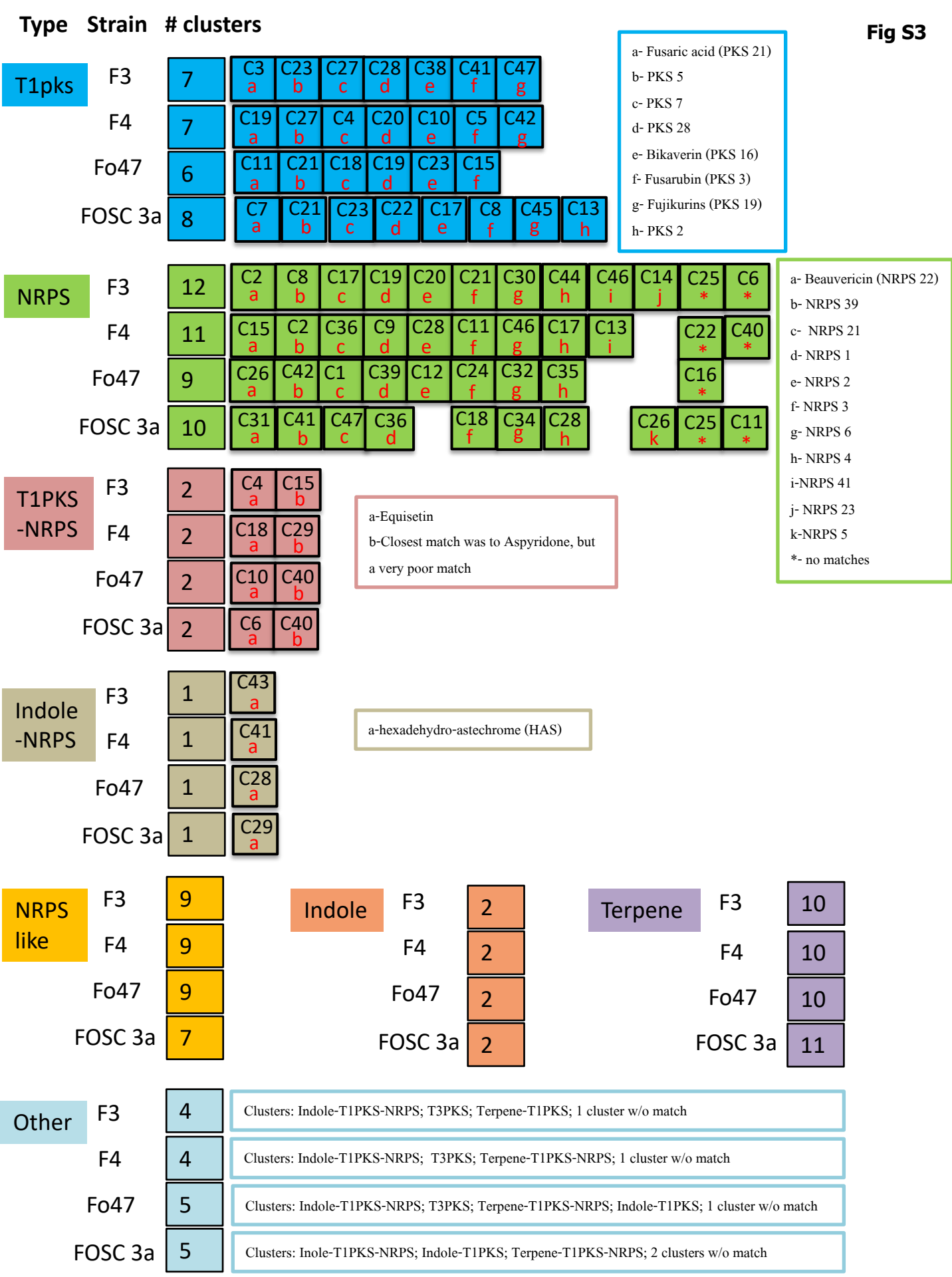

Supplement: FIG S3 [file mSystems.00345-18-sf003.pdf]

**Fig S4**

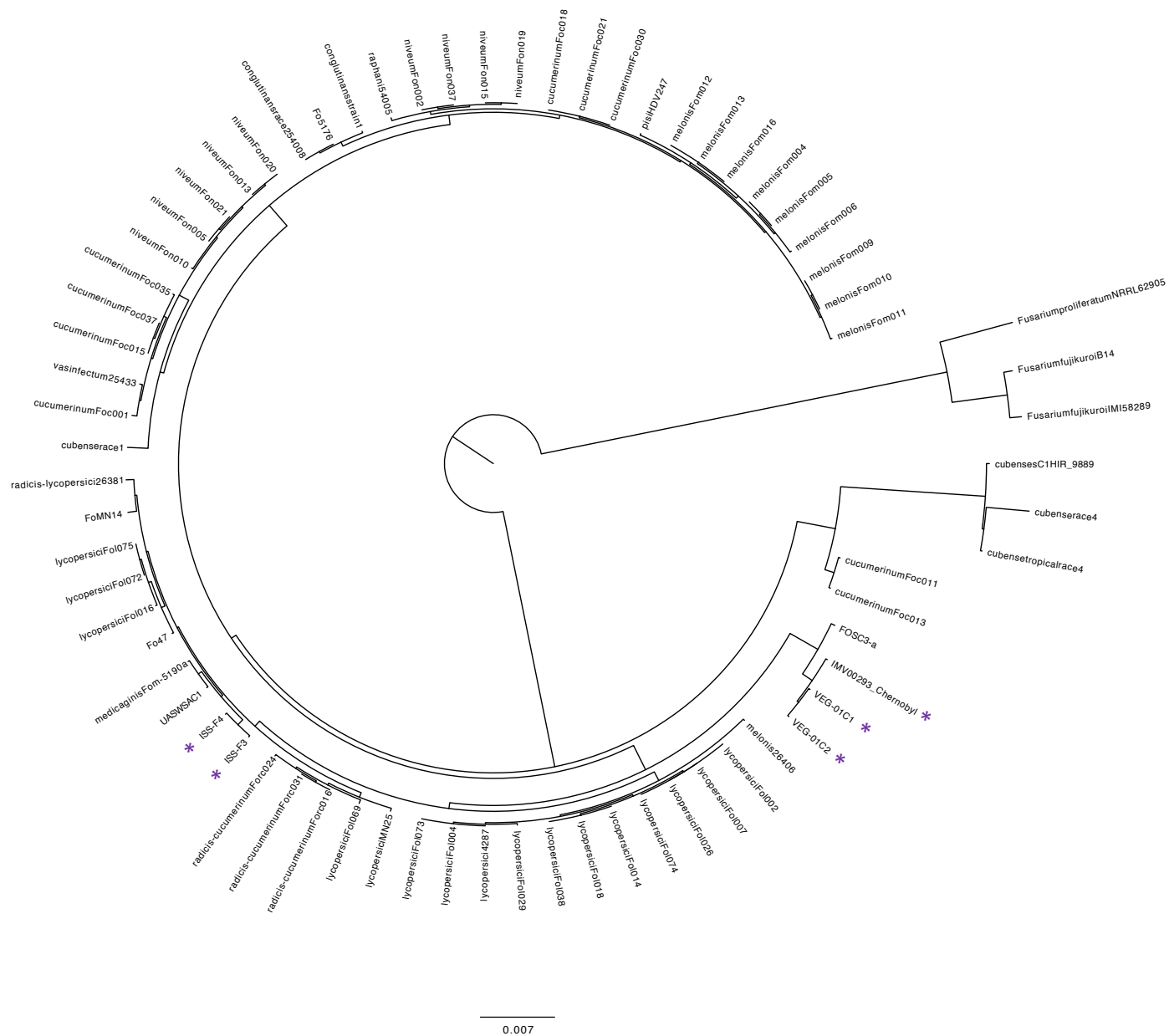

Supplement: FIG S4 [file mSystems.00345-18-sf004.pdf]
