# Supplementary material for: Genomic Characterization and Virulence Potential of Two Fusarium oxysporum Isolates Cultured from the International Space Station
Source: mSystems. 2019 Mar 19;4(2):e00345-18. doi: 10.1128/mSystems.00345-18 (PMC6426649; doi:10.1128/mSystems.00345-18)
Supplement: FIG S2 [file mSystems.00345-18-sf002.pdf]

Fig S2

A

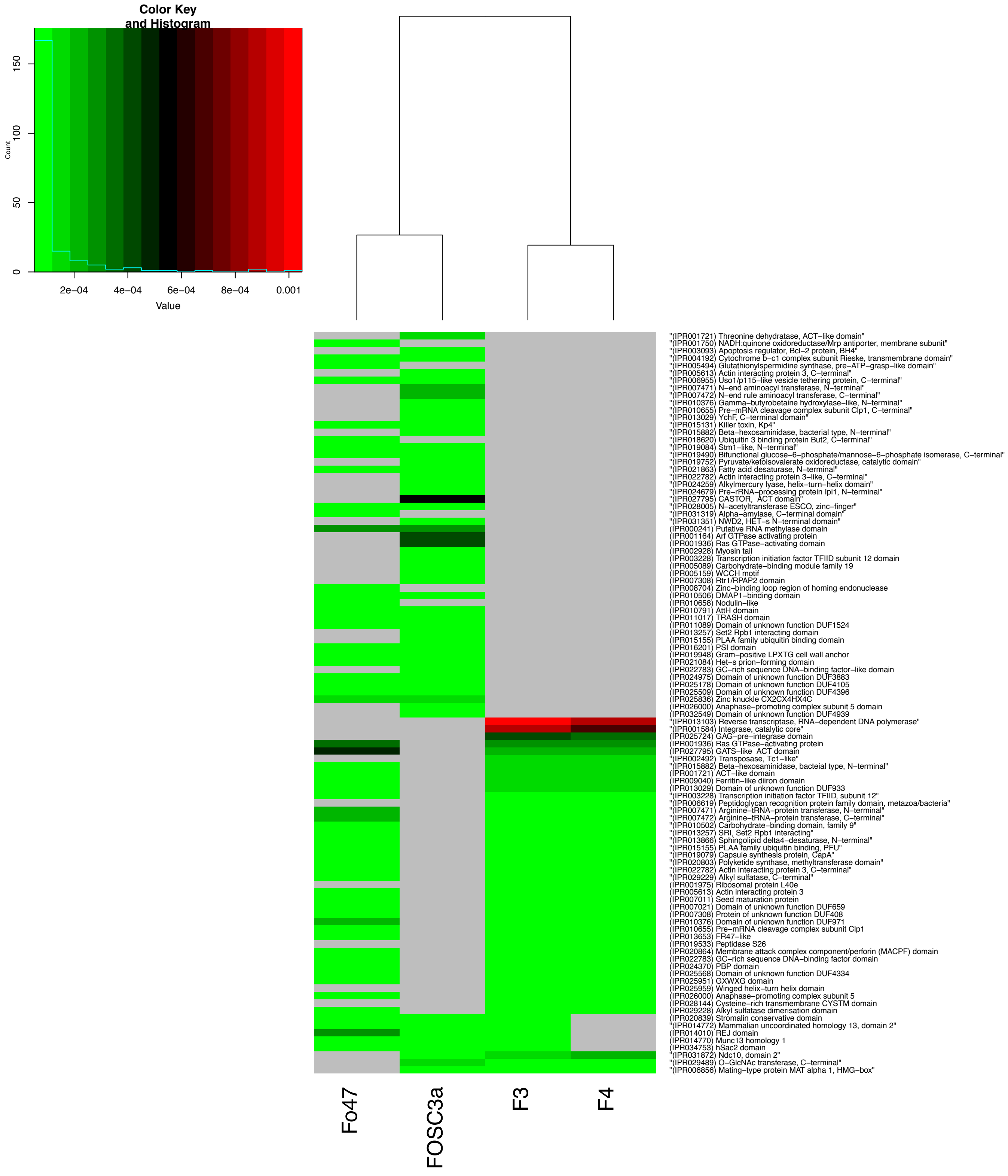
Color Key
and Histogram
Count
0
50
100
150
2e−04
4e−04
6e−04
8e−04
0.001
Value
"(IPR001721) Threonine dehydratase, ACT−like domain"
"(IPR001750) NADH:quinone oxidoreductase/Mrp antiporter, membrane subunit"
"(IPR003093) Apoptosis regulator, Bcl−2 protein, BH4"
"(IPR004192) Cytochrome b−c1 complex subunit Rieske, transmembrane domain"
"(IPR005494) Glutathionylspermidine synthase, pre−ATP−grasp−like domain"
"(IPR005613) Actin interacting protein 3, C−terminal"
"(IPR006955) Uso1/p115−like vesicle tethering protein, C−terminal"
"(IPR007471) N−end aminoacyl transferase, N−terminal"
"(IPR007472) N−end rule aminoacyl transferase, C−terminal"
"(IPR010376) Gamma−butyrobetaine hydroxylase−like, N−terminal"
"(IPR010655) Pre−mRNA cleavage complex subunit Clp1, C−terminal"
"(IPR013029) YchF, C−terminal domain"
"(IPR015131) Killer toxin, Kp4"
"(IPR015882) Beta−hexosaminidase, bacterial type, N−terminal"
"(IPR018620) Ubiquitin 3 binding protein But2, C−terminal"
"(IPR019084) Stm1−like, N−terminal"
"(IPR019490) Bifunctional glucose−6−phosphate/mannose−6−phosphate isomerase, C−terminal"
"(IPR019752) Pyruvate/ketoisovalerate oxidoreductase, catalytic domain"
"(IPR021863) Fatty acid desaturase, N−terminal"
"(IPR022782) Actin interacting protein 3−like, C−terminal"
"(IPR024259) Alkylmercury lyase, helix−turn−helix domain"
"(IPR024679) Pre−rRNA−processing protein Ipi1, N−terminal"
"(IPR027795) CASTOR, ACT domain"
"(IPR028005) N−acetyltransferase ESCO, zinc−finger"
"(IPR031319) Alpha−amylase, C−terminal domain"
"(IPR031351) NWD2, HET−s N−terminal domain"
(IPR000241) Putative RNA methylase domain
(IPR001164) Arf GTPase activating protein
(IPR001936) Ras GTPase−activating domain
(IPR002928) Myosin tail
(IPR003228) Transcription initiation factor TFIID subunit 12 domain
(IPR005089) Carbohydrate−binding module family 19
(IPR005159) WCCH motif
(IPR007308) Rtr1/RPAP2 domain
(IPR008704) Zinc−binding loop region of homing endonuclease
(IPR010506) DMAP1−binding domain
(IPR010658) Nodulin−like
(IPR010791) AttH domain
(IPR011017) TRASH domain
(IPR011089) Domain of unknown function DUF1524
(IPR013257) Set2 Rpb1 interacting domain
(IPR015155) PLAA family ubiquitin binding domain
(IPR016201) PSI domain
(IPR019948) Gram−positive LPXTG cell wall anchor
(IPR021084) Het−s prion−forming domain
(IPR022783) GC−rich sequence DNA−binding factor−like domain
(IPR024975) Domain of unknown function DUF3883
(IPR025178) Domain of unknown function DUF4105
(IPR025509) Domain of unknown function DUF4396
(IPR025836) Zinc knuckle CX2CX4HX4C
(IPR026000) Anaphase−promoting complex subunit 5 domain
(IPR032549) Domain of unknown function DUF4939
"(IPR013103) Reverse transcriptase, RNA−dependent DNA polymerase"
"(IPR001584) Integrase, catalytic core"
(IPR025724) GAG−pre−integrase domain
(IPR001936) Ras GTPase−activating protein
(IPR027795) GATS−like ACT domain
"(IPR002492) Transposase, Tc1−like"
"(IPR015882) Beta−hexosaminidase, bacteial type, N−terminal"
(IPR001721) ACT−like domain
(IPR009040) Ferritin−like diiron domain
(IPR013029) Domain of unknown function DUF933
"(IPR003228) Transcription initiation factor TFIID, subunit 12"
"(IPR006619) Peptidoglycan recognition protein family domain, metazoa/bacteria"
"(IPR007471) Arginine−tRNA−protein transferase, N−terminal"
"(IPR007472) Arginine−tRNA−protein transferase, C−terminal"
"(IPR010502) Carbohydrate−binding domain, family 9"
"(IPR013257) SRI, Set2 Rpb1 interacting"
"(IPR013866) Sphingolipid delta4−desaturase, N−terminal"
"(IPR015155) PLAA family ubiquitin binding, PFU"
"(IPR019079) Capsule synthesis protein, CapA"
"(IPR020803) Polyketide synthase, methyltransferase domain"
"(IPR022782) Actin interacting protein 3, C−terminal"
"(IPR029229) Alkyl sulfatase, C−terminal"
(IPR001975) Ribosomal protein L40e
(IPR005613) Actin interacting protein 3
(IPR007011) Seed maturation protein
(IPR007021) Domain of unknown function DUF659
(IPR007308) Protein of unknown function DUF408
(IPR010376) Domain of unknown function DUF971
(IPR010655) Pre−mRNA cleavage complex subunit Clp1
(IPR013653) FR47−like
(IPR019533) Peptidase S26
(IPR020864) Membrane attack complex component/perforin (MACPF) domain
(IPR022783) GC−rich sequence DNA−binding factor domain
(IPR024370) PBP domain
(IPR025568) Domain of unknown function DUF4334
(IPR025951) GXWXG domain
(IPR025959) Winged helix−turn helix domain
(IPR026000) Anaphase−promoting complex subunit 5
(IPR028144) Cysteine−rich transmembrane CYSTM domain
(IPR029228) Alkyl sulfatase dimerisation domain
(IPR020839) Stromalin conservative domain
"(IPR014772) Mammalian uncoordinated homology 13, domain 2"
(IPR014010) REJ domain
(IPR014770) Munc13 homology 1
(IPR034753) hSac2 domain
"(IPR031872) Ndc10, domain 2"
"(IPR029489) O−GlcNAc transferase, C−terminal"
"(IPR006856) Mating−type protein MAT alpha 1, HMG−box"
Fo47
FOSC3a
F3
F4

B

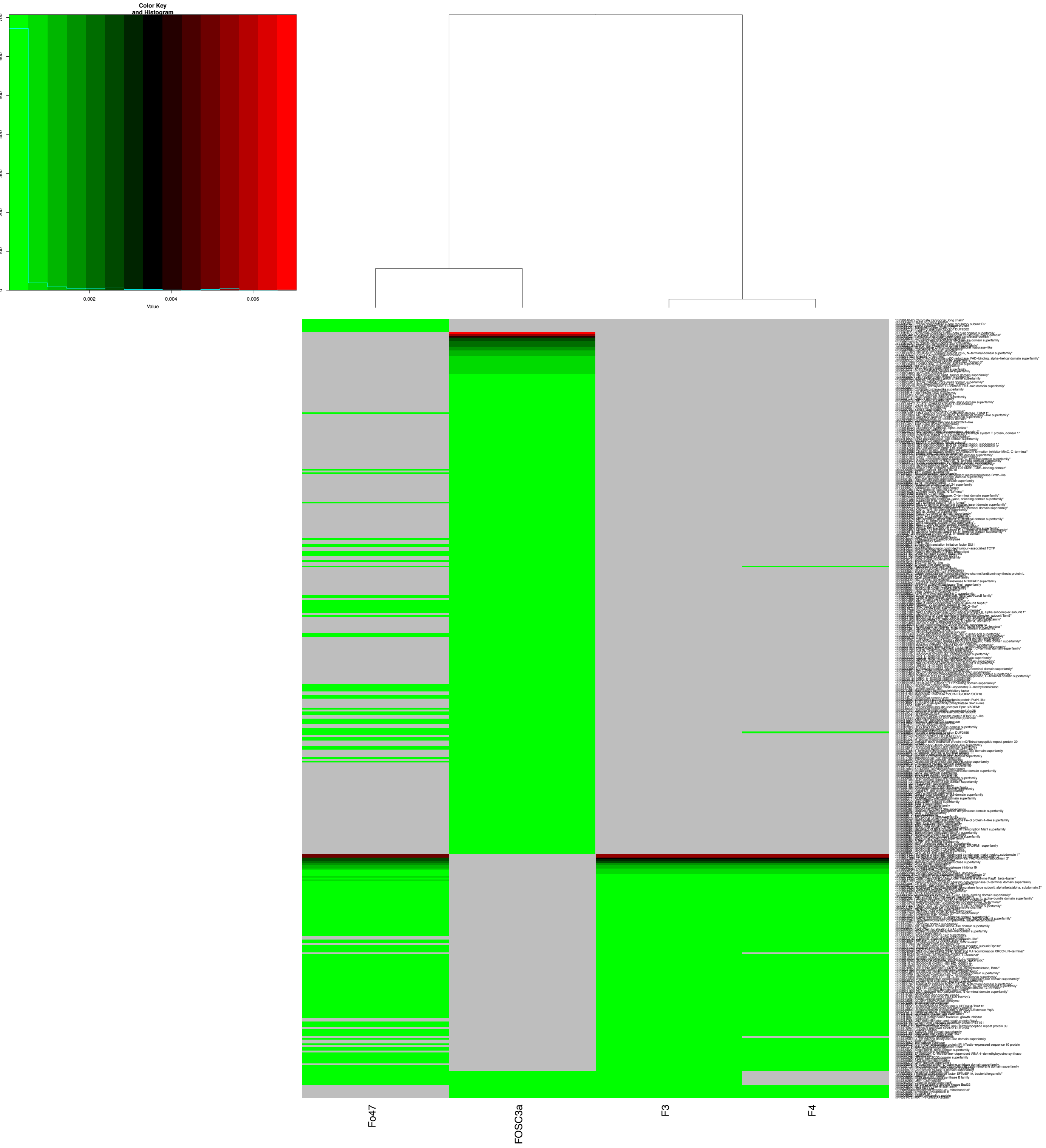
Color Key
and Histogram
0.002
0.004
0.006
Value
Fo47
FOSC3a
F3
F4
